# Motor Imagery Cognitive Network after Left Ischemic Stroke: Study of the Patients during Mental Rotation Task

**DOI:** 10.1371/journal.pone.0077325

**Published:** 2013-10-22

**Authors:** Jing Yan, Junfeng Sun, Xiaoli Guo, Zheng Jin, Yao Li, Zhijun Li, Shanbao Tong

**Affiliations:** 1 School of Biomedical Engineering, Shanghai Jiao Tong University, Shanghai, China; 2 Med-X Research Institute, Shanghai Jiao Tong University, Shanghai, China; 3 Department of Neurology, The Fifth People’s Hospital of Shanghai, Shanghai, China; 4 Department of Automation, School of Electronic Information and Electrical Engineering, Shanghai Jiao Tong University, Shanghai, China; University of Bologna, Italy

## Abstract

Although motor imagery could improve motor rehabilitation, the detailed neural mechanisms of motor imagery cognitive process of stroke patients, particularly from functional network perspective, remain unclear. This study investigated functional brain network properties in each cognitive sub-stage of motor imagery of stroke patients with ischemic lesion in left hemisphere to reveal the impact of stroke on the cognition of motor imagery. Both stroke patients and control subjects participated in mental rotation task, which includes three cognitive sub-stages: visual stimulus perception, mental rotation and response cognitive process. Event-related electroencephalograph was recorded and interdependence between two different cortical areas was assessed by phase synchronization. Both global and nodal properties of functional networks in three sub-stages were statistically analyzed. Phase synchronization of stroke patients significantly reduced in mental rotation sub-stage. Longer characteristic path length and smaller global clustering coefficient of functional network were observed in patients in mental rotation sub-stage which implied the impaired segregation and integration. Larger nodal clustering coefficient and betweenness in contralesional occipitoparietal and frontal area respectively were observed in patients in all sub-stages. In addition, patients also showed smaller betweenness in ipsilesional central-parietal area in response sub-stage. The compensatory effects on local connectedness and centrality indicated the neuroplasticity in contralesional hemisphere. The functional brain networks of stroke patients demonstrated significant alterations and compensatory effects during motor imagery.

## Introduction

Motor imagery has been employed in neurological rehabilitation to improve the overall behavior performance after stroke [1]–[2]. During motor imagery, the representation of a specific motor action is implicitly activated within working memory, but without an overt motor output [3]. Posterior parietal cortex, premotor and supplementary motor areas, primary motor cortex, and subcortical basal ganglia were activated during motor imagery [4]–[5]. All these structures were involved in motor planning and execution, and their activation during motor imagery suggested that actual and mentally simulated movements largely share the similar cerebral structures [6]. Most previous studies focused on the excitatory or inhibition of some focal brain structures during motor imagery [4]–[5]. However, the interactions among different brain structures were crucial for cognition, and, brain is organized according to the fundamental principle of functional segregation and integration, that is, local cortical regions are specified for certain functions, while spatially separated cortices are integrated by sparse long distance neural connectivity to achieve higher-order cognitive function [7]. A brain network consists of a set of nodes (e.g., single neuron, neural ensemble, anatomical brain areas) and edges (e.g., anatomical connection between neural elements in structural brain network, or statistically temporal association between neural signals in functional brain network). A cohort of parameters (e.g., node degree, nodal clustering coefficient and betweenness, global clustering coefficient, and characteristic path length) could be employed to characterize the brain network properties at either nodal or global scale [8]–[10].

Until now, the detailed neural mechanisms of motor imagery of stroke patients remain unclear since few studies focused on its cognitive process. Our previous study, to the best of our knowledge, was the only report on the cognitive process of motor imagery of stroke [11]. We used a mental rotation task (MRT) that requires subject to perform identification and laterality judgment (e.g., left or right) of the presented body part picture which was rotated to different spatial angles [4], [12], [13], and investigated the neural mechanisms of motor imagery of stroke patients from aspects of behavior and event-related oscillation [11]. It indicated the impairment of behavior response for stroke patients (i.e., longer response time and lower accuracy rate compared to normal control subjects) and the cognitive progress of MRT at least includes three sub-stages: (i) visual stimulus perceptual encoding (i.e., identification of stimulus and its orientation, 0–300 ms), (ii) mental rotation (300–800 ms), and (iii) response (after 800 ms) [11]. In this study, functional brain networks of stroke patients in each cognitive sub-stage were examined from both global and nodal perspectives. This study is intended understanding the cognitive consequence of stroke in the aspects of cortical segregation and integration.

## Materials and Methods

### Subjects

Eleven stroke patients (mean age: 60.3±12.8 years; male/female = 9/2) with ischemic stroke lesion in left hemisphere (lesion details were presented in Table 1) were recruited from the Department of Neurology in the Fifth People’s Hospital of Shanghai. All patients suffered a moderate stroke (NIHSS: range from 6–10) with similar symptoms of hemiplegic paralysis. None of them had deficit in using right index finger to press the response button. Eleven age-matched (p = 0.49 in t-test) healthy subjects (mean age: 60.1±6.9 years; male/female = 6/5) were recruited as control group. All control subjects reported no history of neurological diseases or psychiatric disorders. All participants were right-handed with normal or correct-to-normal vision and signed a written informed consent to participate in accordance with the Declaration of Helsinki. This study was approved by Ethic Committees of Shanghai Jiao Tong University and the Fifth People’s Hospital of Shanghai.

**Table 1:** Subjects demography.

Patient No.	Age(y)/Sex	Post-stroke (months)	NIHSS	Lesion location	ControlNo.	Age(y)/Sex
P1	74/M	6	9	Parietal lobe, basal ganglia	C1	54/M
P2	53/M	9	10	Frontal lobe	C2	62/M
P3	45/M	8	8	Frontal, parietal lobe	C3	45/F
P4	46/F	12	8	Parietal lobe	C4	57/F
P5	66/M	4	7	Parietal lobe	C5	60/F
P6	64/M	5	9	Frontal lobe, basal ganglia	C6	67/F
P7	57/M	7	8	Frontal, parietal lobe	C7	56/M
P8	55/F	6	6	Frontal lobe	C8	67/M
P9	80/M	14	6	Frontal lobe, basal ganglia	C9	66/F
P10	46/M	9	7	Frontal, parietal lobe	C10	60/M
P11	77/M	10	8	Parietal lobe, basal ganglia	C11	68/M

M = Male; F = Female; y = years; NIHSS = National Institutes of Health Stroke Scale.

### Stimuli and Experiment Procedure

**Figure 1:**
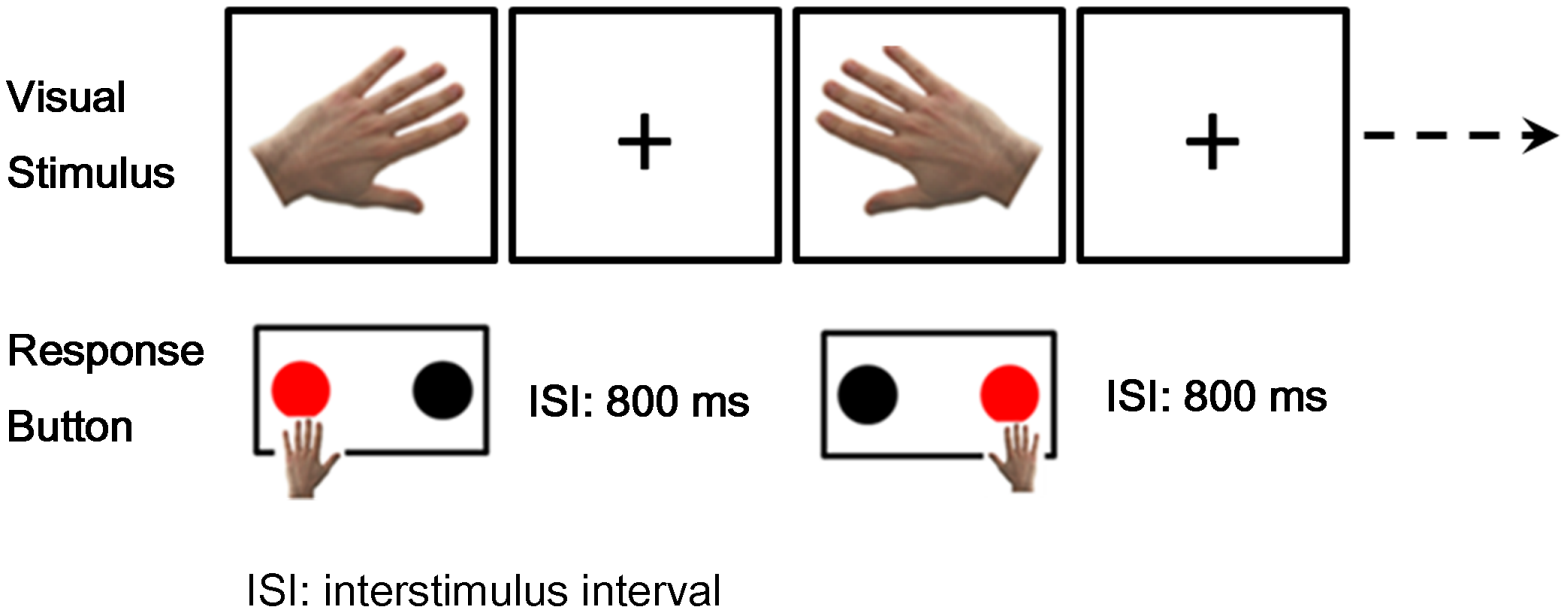
The experimental diagram of mental rotation task (MRT). Red wafer indicates the correct response to the corresponding stimulus. The inter-stimulus interval was a crosshair lasting for 800 ms.

Figure 1 presented the schematic diagram of the experiment. During the experiment, stimulus pictures of right or left hand at different angles were randomly presented on the screen with viewing distance about 50 cm and visual angle about 2.5° in height. In total, there were 12 [2 HAND (left and right hand) × 6 ANGLE (0°, 60°, 120°, 180°, 240° and 300°)] types of stimuli in this study. In each block, 96 stimuli (48 left hand and 48 right hand pictures) were adopted with probabilities: 0°, 25%; 60°, 12.5%; 120°, 12.5%; 180°, 25%; 240°, 12.5%; 300°, 12.5% respectively. There were six blocks for control subjects while two blocks for stroke patients as they are more likely to be suffered in a longer experiment. During interstimulus interval (ISI), a black crosshair was presented for 800 ms. Hand pictures were presented until participants pressed the response button. Subjects were asked to keep minimal head and eye movements during the experiments. They were requested to press the left button using left index finger for left hand stimuli and the right button using right index finger for right hand stimuli as quickly and accurately as possible. Subjects took 3–5 minutes rest between blocks. All stimulation procedures were controlled by E-Prime (version 2.0, Psychology Software Tools Inc, Pittsburgh, USA).

### EEG Recording and Data Acquisition

EEG signals were continuously recorded using Brain Vision Recorder (version 1.03, Brain Products GmbH, Munich, Germany) from 32 Ag-AgCl electrodes (EasyCap, Brain Products GmbH, Munich, Germany) at a sampling rate of 1000 Hz and impedance below 5 kΩ at each electrode. The electrode FCz served as the default reference, and EEG signals were further offline re-referenced to the average of the electrodes at the left and right mastoids. Horizontal and vertical electrooculograms (EOGs) were also recorded for rejecting artifacts due to eye movements. EOG contaminations in EEG were removed by independent component analysis implemented in Brain Vision Analyzer (version 2.0, Brain Products GmbH, Munich, Germany) [14]. After re-reference and EOG removal, there were total 28 channels of EEG data left for further analysis.

### EEG Data Preprocessing

EEG preprocessing was performed offline using Brain Vision Analyzer (Brain Products GmbH, Munich, Germany). Only EEG signals for correctly responded stimuli were included in subsequent analysis. Artifact-free EEG signals were segmented into trials of 1200 ms each starting from the onset of stimulus picture. All trials were further filtered into beta band (13–30 Hz), which was reported to be relevant to motor cognition [15]–[18]. Our previous study of the same EEG dataset has showed significant EEG oscillations (i.e., P200 in 0–300 ms, P300 in 300–800 ms, and late component after 800 ms), which were specific to the cognitive sub-states of mental rotation task [11]. To examine the alterations of functional brain network in different cognitive sub-stages, each trial was divided into three subsegments, i.e., 0–300 ms (Beginning), 300–800 ms (Middle), and 800–1200 ms (End) corresponding to visual stimulus encoding, mental rotation, and response sub-stage respectively [11]–[12]. Note that for the EEG data used in this study, another study focused on behavior and EEG oscillations has been published [11]. In this paper, spontaneous EEG data in different time periods were analyzed from the perspective of cognitive network.

### Phase Synchronization Analysis

Phase synchronization (PS) analysis has been commonly used to quantify the association between neural oscillations and examine the large-scale integration of neural activity [19]–[20]. For an epoch of real-value narrow-band EEG signal 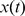, its analytic signal is defined as
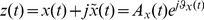
(1)where
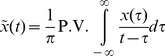
(2)is the Hilbert transform of 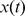 (here, P.V. means that the integral is taken in the sense of Cauchy principal value). 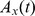 and
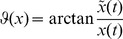
(3)are the instantaneous amplitude and instantaneous phase (IP) of signal 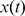 respectively. Let 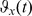 and 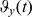 denote the IPs of two signals 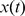 and 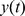 measured from two coupled variables or systems (i.e., two EEG channels in this study). Then if the relationship
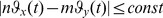
(4)holds for a period of time, that is, the difference between IPs is bounded with respect to time, the coupled variables or systems are said to be in 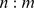 PS, where 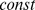 denotes a constant, 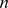 and 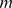 are two positive integers. Most neural electrophysiological signals are of the case of 1∶1 PS, which was also adopted in this study [21]–[23]. To evaluate the level of PS, various phase synchronization index (PSI) have been proposed. Among them, the mean phase coherence (MPC) is commonly used, which is defined as
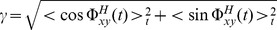
(5)where 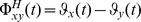 is the IP difference, and 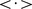 denotes the average of variable over time. The value of 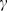 (i.e., PSI) is among [0, 1], with 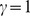 implying perfect phase locking between 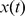 and 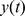, and 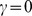 indicating no PS at all. PS analysis only takes the IPs of signals into consideration and excludes the effect of amplitude, thus it could detect weak interaction between two EEG signals that might be overlooked by other measures of interdependence.

In each cognitive sub-stage, a 28-by-28 averaged associate matrix 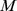 was constructed by averaging all matrices of the same stimulus type for each subject respectively. Each element in 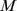 was corresponding to the PSI of each pair of EEG channels. Thus, for each subject, there were 12 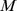
s corresponding to 12 stimuli types in each cognitive sub-stage for further statistical analysis. In addition, according to the location of electrodes, the following three classes of PSIs of 12 stimuli types were examined in each cognitive sub-stage: (1) the left intra-hemispheric PSI (denoted by PSI_L_), which was the average of all the interactions within the left hemisphere; (2) the right intra-hemispheric PSI (denoted by PSI_R_), which was the average of all the interactions within the right hemisphere; and (3) the inter-hemispheric PSI (denoted by PSI_I_), which was the average of all the interactions between two hemispheres.

### Network Analysis

With a preset threshold 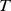, an associate matrix 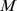 could be converted into an adjacent matrix 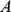 by setting the entry of 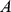 to be 1 if the associate strength of the corresponding entry in 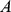 is greater than 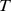, or otherwise to be 0. To construct the weighted network, a weighted matrix 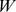 could be generated by assigning the values of the entries in associate matrix 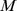 to the corresponding entries of 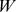 if their values are greater than 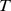, and setting the values of the rest entries to be 0. Several graph parameters were used to characterize the topological features of the network. The nodal parameters were calculated for each node, i.e., nodal clustering coefficient (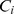) and betweenness (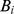), quantifying the nodal properties of network. In addition, clustering coefficient (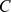), characteristic path length (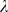) and small-worldness index (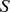), were used to describe the global properties of network [9], [24]–[25].

For weighted network with 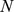 nodes (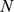 = 28 in this study), the clustering coefficient of each node is defined as
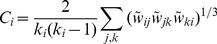
(6)where 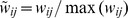 is the weight scaled by the largest weight in the network. The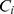 of a node indicates how densely its neighbors are connected [8]. The clustering coefficient (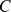) of a network is then defined as the average of all nodal clustering coefficients, that is,



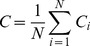
(7)The clustering coefficient of network indicates the connectedness among the neighbors of the same node in an average statistical sense [26]. For brain network analysis, this parameter could be used as a measure of segregation of neural organization from global perspective.

In analysis of weighted brain network, the path length between node 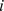 and 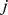 is defined as the sum of the reciprocals of the weights for the edges that could link these two nodes, and the shortest path length 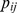 is the minimum one among all path lengths between node 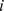 and 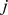. The characteristic path length (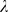) of a network is defined as the mean of the shortest path lengths between all possible pairs of nodes, that is,
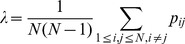
(8)


The characteristic path length (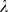) is an indicator of typical separation between two nodes over the whole network [7]. For brain network analysis, this parameter can quantify the integration of neural organization from global prospective.

In weighted network, betweenness (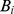) of node 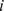 is defined as
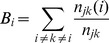
(9)where 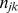 is the total number of the shortest path lengths between node 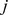 and 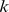, and 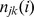 is the total number of the shortest path lengths that link node 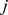 and 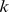 and pass through node 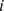 as well. The betweenness centrality of a node indicates its role and importance in the information transfer over the whole network [8], [27].

To evaluate the alteration of small-world property (i.e., network with large clustering coefficient while short characteristic path length) of brain network after stroke, the clustering coefficient (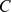) and characteristic path length (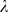) of brain network were compared with the corresponding metrics of twenty size-matched random networks. A metric called small-worldness index (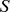) is defined as
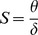
(10)where 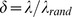, 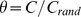, 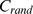 and 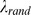 are the means of the clustering coefficients and the characteristic path lengths of all random networks respectively. The value of the small-worldness index is greater than 1 for small-world network [7].

The threshold 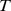 means the number of largest edges the network has in this study. We employed different thresholds, resulting in networks with different numbers of largest edges from 90 to 180 for the whole cognitive process (0–1200 ms), to examine the relationship between global network properties (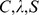) and the value of threshold. After that, both global (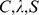) and nodal (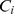 and 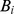) parameters of network with a specifically chosen threshold in each cognitive sub-stage were statistically analyzed to reveal the functional cognitive network alterations after stroke.

### Statistical Analysis

Repeated measures ANOVA (analysis of variance) were used to test the statistical significance in this study. GROUP (Controls vs. Patients) was between-subjects factor for all statistical analysis. For ANOVA analysis of PSIs in each cognitive sub-stage, HEMISPHERE (Left-Hemisphere vs. Right-Hemisphere vs. Inter-Hemisphere), ANGLE (0°, 60°, 120°, 180°, 240°, and 300°) and HAND (Left Hand vs. Right Hand) were within-subjects factors. For ANOVA analysis of global network parameters (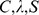), HAND and ANGLE were used as within-subjects factors. HAND, ANGLE and CHANNEL (28 EEG channels) were used as within-subjects factors in ANOVA analysis of nodal network parameters (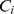 and 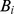). In addition, post hoc t-tests were performed to the overall global network properties (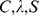) with different thresholds 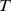. False discovery rate (FDR, q<0.05) was applied for multiple comparison corrections [28]. Note that ANOVA analysis and t-tests were performed using SPSS version 17 (SPSS, Chicago, IL).

## Results

### Results of PSIs

**Figure 2:**
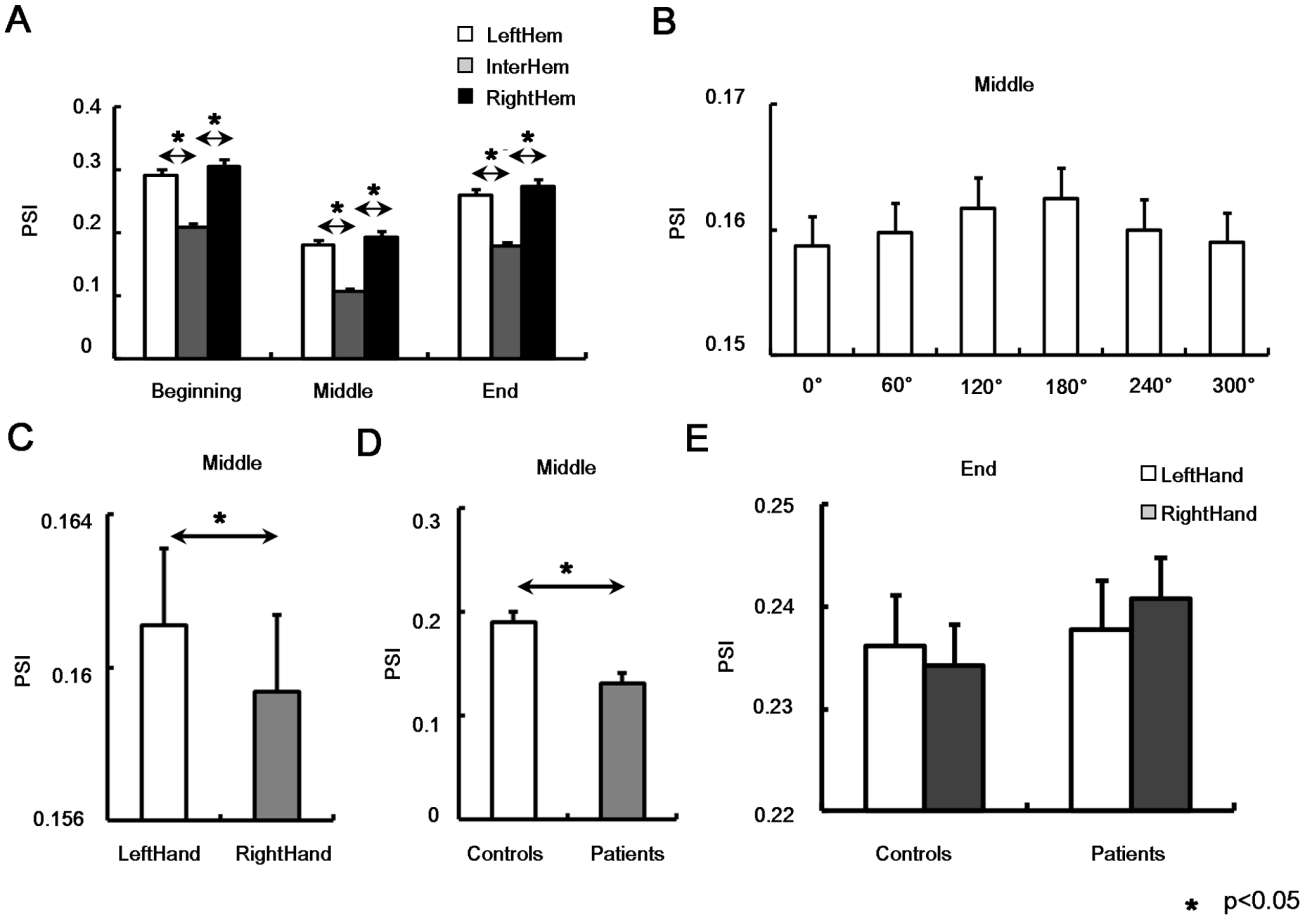
Results of phase synchronization index (PSI). **A:** PSI with respect to hemisphere factor in three sub-stages were shown; PSI with respect to ANGLE (**B**), HAND (**C**) and GROUP (**D**) factor in Middle sub-stage were illustrated; **E:** Interaction effect of GROUP and HAND factor on PSI in End sub-stage were shown. Significant difference was indicated by *(p<0.05).

ANOVA results of PSIs were shown in Table 2. In Beginning sub-stage, only HEMISPHERE showed significant main effect (F(2,40) = 112.031, p<0.001). Inter-hemispheric PSIs (PSI_I_) were significantly smaller than intra-hemispheric PSIs (PSI_L_ and PSI_R_) during visually encoding the stimulus (Fig. 2A).

**Table 2:** ANOVA analysis of phase synchronization index (PSI).

	Beginning(0–300 ms)	Middle(300–800 ms)	End(800–1200 ms)
**GROUP**	F(1,20) = 0.280, p = 0.602	F(1,20) = 16.333, p = 0.001*	F(1,20) = 0.056, p = 0.816
**HEMISPHERE**	F(2,40) = 112.031, p<0.001**	F(2,40) = 88.057, p<0.001**	F(2,40) = 85.797, p<0.001**
**HAND**	F(1,20) = 3.624, p = 0.071	F(1,20) = 4.718, p = 0.042*	F(1,20) = 0.228, p = 0.638
**ANGLE**	F(5,100) = 0.815, p = 0.542	F(5,100) = 2.492, p = 0.036*	F(5,100) = 0.857, p = 0.513
**GROUP × HEMISPHERE**	F(2,40) = 1.094, p = 0.345	F(2,40) = 0.316, p = 0.731	F(2,40) = 0.419, p = 0.660
**GROUP × HAND**	F(1,20) = 1.174, p = 0.291	F(1,20) = 0.082, p = 0.778	F(1,20) = 4.601, p = 0.044*
**GROUP × ANGLE**	F(5,100) = 0.815, p = 0.542	F(5,100) = 1.119, p = 0.355	F(5,100) = 2.005, p = 0.084

Significance was indicated by *(p<0.05) and **(p<0.001).

In Middle sub-stage, GROUP showed significant main effect that stroke patients had significantly smaller PSI value than control subjects (F(1,20) = 16.333, p = 0.001) (Fig. 2D). Furthermore, PSI_I_ was smaller than PSI_L_ and PSI_R_ indicating the HEMISPHERE main effect in this sub-stage (F(2,40) = 88.057, p<0.001) (Fig. 2A). Significant main effect of HAND was observed (F(1,20) = 4.718, p = 0.042), i.e., PSI value for right hand was significantly smaller than that for left hand in this sub-stage (Fig. 2C). In addition, ANGLE factor showed significant “angle effect” of PSI in this sub-stage (F(5,100) = 2.492, p = 0.036), i.e., PSI increased with angle and reached maximum at 180° (Fig. 2B). No significant interaction between GROUP and other within-subjects factors were observed (all, p>0.355).

In End sub-stage, HEMISPHERE factor showed significant main effect (F(2,40) = 85.797, p<0.001), which was also because PSI_I_ were significantly smaller than PSI_L_ and PSI_R_ (Fig. 2A). In addition, significant interaction between GROUP and HAND was observed (F(1,20) = 4.601, p = 0.044), since control subjects had smaller PSI for right hand than that for left hand in this sub-stage, while stroke patients showed smaller PSI for left (unaffected) hand than that for right (affected) hand (Fig. 2E). Other factors and interactions didn’t show significant main effect in this sub-stage (all, p>0.084).

In short, phase synchronizations in stroke patients were significantly impaired particularly when patients mentally rotated the hand pictures. During response cognitive process, patients showed larger phase synchronization for affected hand than unaffected hand.

### Results of Global Network Parameters

**Figure 3:**
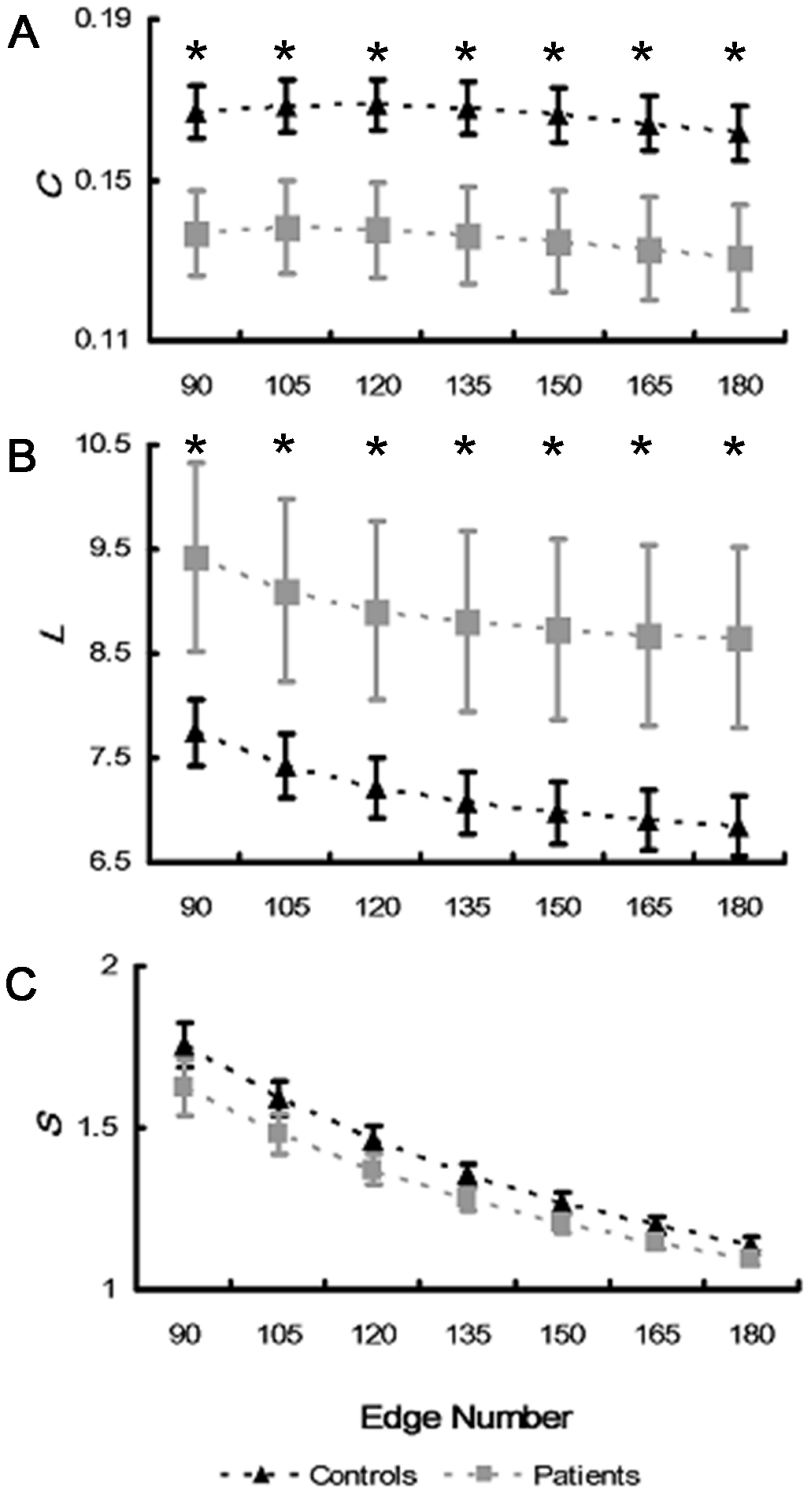
Global network parameters with respect to different thresholds. Global clustering coefficients (**A**), characteristic path lengths (**B**) and small-worldness indexes (**C**) of brain networks during whole MRT (0–1200 ms) with respect to different thresholds were illustrated, respectively. Symbols *indicate the cases of significance difference after multiple comparisons correction by FDR (i.e., with p-values less than the significance threshold estimated by FDR, q<0.05).

Statistical t-test of overall global network properties (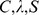), averaged over all 12 stimulus types during whole cognitive process (0–1200 ms), were performed between control group and patient group for seven different thresholds (i.e., 90, 105, 120, 135, 150, 165,180 largest weighted edges in network) respectively (Fig. 3). For all selected thresholds, patient group consistently showed significantly smaller clustering coefficient (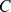) and longer characteristic path length (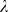) after multiple comparison corrections by FDR (q<0.05) (Fig. 3A–B). In addition, stroke patients had smaller small-worldness index (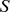), but not significantly (Fig. 3C). All these results indicated that the differences of network properties between patients and controls were consistent under different thresholds. Therefore, network with 90 largest weighted edges would be investigated in each sub-stage in two groups hereafter.

#### Clustering coefficient

**Figure 4:**
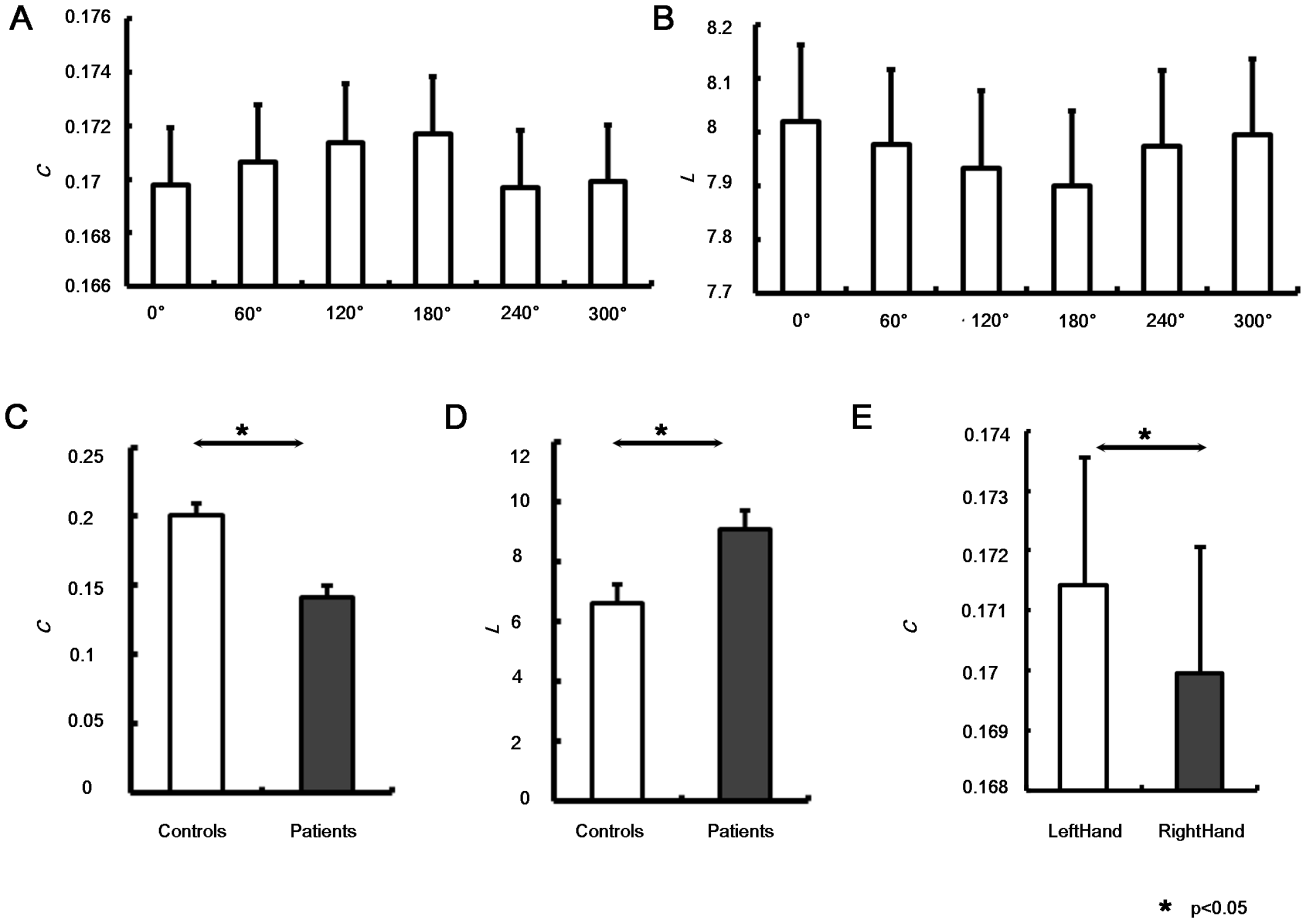
Global network properties in Middle sub-stage. Clustering coefficient (**A**) and characteristic path length (**B**) with respect to ANGLE were shown. GROUP effect on clustering coefficient (**C**) and characteristic path length (**D**) were illustrated. Clustering coefficient in aspect to HAND factor was in (**E**). Significant difference was indicated by *(p<0.05).

Main effects of factors on clustering coefficient (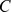) were only observed in Middle sub-stage (Table 3). In Middle sub-stage, GROUP showed significant main effect (F(1,20) = 21.596, p<0.001), which was due to the fact that patients had significantly smaller 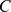 than control subjects (Fig. 4C). Clustering coefficient for right hand was significantly smaller than that for left hand showing HAND main effect (F(1,20) = 5.11, p = 0.035) (Fig. 4E). In addition, significant ANGLE effect was observed (F(5,100) = 2.617, p = 0.029), i.e., 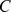 increased with angle and reached maximum at 180° (Fig. 4A). No significant main effect of other factors or their interactions were observed (all, p>0.113).

**Table 3:** ANOVA analysis of global clustering coefficient (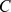).

	Beginning(0–300 ms)	Middle(300–800 ms)	End(800–1200 ms)
**GROUP**	F(1,20) = 0.001, p = 0.971	F(1,20) = 21.596, p<0.001**	F(1,20) = 0.033, p = 0.858
**HAND**	F(1,20) = 4.308, p = 0.051	F(1,20) = 5.11, p = 0.035*	F(1,20) = 0.108, p = 0.746
**ANGLE**	F(5,100) = 1.019, p = 0.410	F(5,100) = 2.617, p = 0.029*	F(5,100) = 1.253, p = 0.290
**GROUP × HAND**	F(1,20) = 0.000, p = 0.992	F(1,20) = 0.006, p = 0.938	F(1,20) = 2.903, p = 0.104
**GROUP × ANGLE**	F(5,100) = 1.371, p = 0.242	F(5,100) = 0.856, p = 0.113	F(5,100) = 1.281, p = 0.278

Significance was indicated by *(p<0.05) and **(p<0.001).

#### Characteristic path length

ANOVA results of characteristic path length (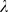) in three sub-stages were listed in Table 4. Similar with clustering coefficient, major statistical significances of characteristic path length were observed in Middle sub-stage. Main effect of GROUP was observed (F(1,20) = 10.242, p = 0.004), i.e., patients had significantly longer characteristic path length than control subjects in Middle sub-stage (Fig. 4D). Furthermore, ANGLE also showed significant main effect (F(5,100) = 2.617, p = 0.029), i.e., characteristic path length (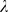) decreased with angle and reached the minimum at 180° (Fig. 4B). No significant main effect of other factors or their interactions were observed (all, p>0.387).

**Table 4:** ANOVA analysis of characteristic path length (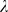).

	Beginning(0–300 ms)	Middle(300–800 ms)	End(800–1200 ms)
**GROUP**	F(1,20) = 0.070, p = 0.793	F(1,20) = 10.242, p = 0.004*	F(1,20) = 0.005, p = 0.947
**HAND**	F(1,20) = 3.685, p = 0.069	F(1,20) = 0.015, p = 0.904	F(1,20) = 2.016, p = 0.171
**ANGLE**	F(5,100) = 0.285, p = 0.921	F(5,100) = 2.617, p = 0.029*	F(5,100) = 2.227,p = 0.057
**GROUP × HAND**	F(1,20) = 0.066, p = 0.800	F(1,20) = 0.781, p = 0.387	F(1,20) = 5.825, p = 0.026
**GROUP × ANGLE**	F(5,100) = 1.185, p = 0.322	F(5,100) = 0.177, p = 0.971	F(5,100) = 1.794,p = 0.121

Significance was indicated by *(p<0.05).

In short, global clustering coefficient and characteristic path length results showed significant “angle effect” in both groups. However, patients had significantly smaller clustering coefficient and longer characteristic path length particularly when they mentally rotated the hand pictures.

### Results of Nodal Network Parameters

To assess the alterations of nodal network properties after stroke, nodal clustering coefficient (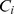) and betweenness (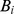) of network with 90 largest weighted edges were investigated in each sub-stage in both groups. Topographic mappings of nodal network parameters were presented using a revised topoplot algorithm from EEGLAB [29].

#### Nodal clustering coefficient

**Figure 5:**
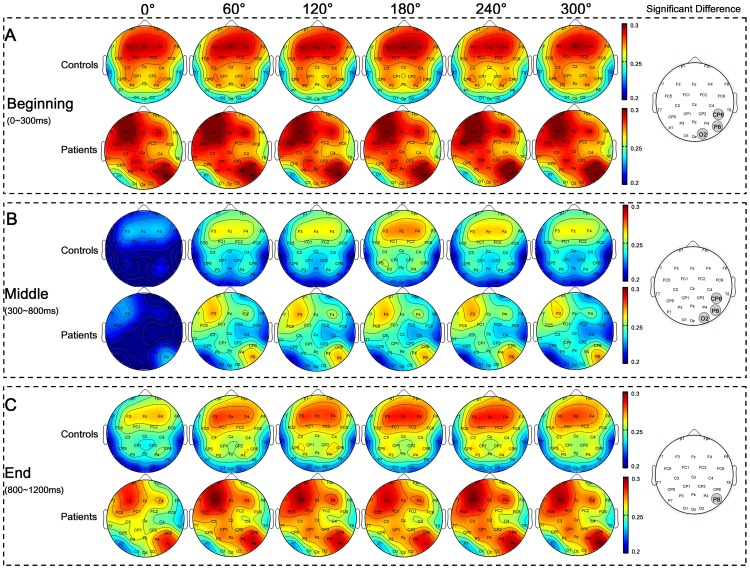
Nodal clustering coefficients in three sub-stages. Nodal clustering coefficients of two groups with respect to angle in Beginning (**A**), Middle (**B**), and End sub-stages (**C**) were illustrated respectively. Gray wafers indicated the channels where stroke patients have larger clustering coefficient than control subjects by t-test (p<0.05). However, the t-test p-values of these channels are greater than the significance threshold estimated by FDR (q<0.05) for multiple comparisons correction.

For ANOVA analysis of nodal clustering coefficient (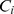), GROUP and HAND did not show significant main effect in all sub-stages (all, p>0.069) (Table 5). Main effect of ANGLE was observed in all sub-stages (all, p<0.001), which was due to the fact that significantly smaller 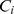 was observed for non-rotated (0°) stimuli than rotated ones (60°, 120°, 180°, 240° and 300°). Such an “angle effect” was more prominent in Middle and End sub-stages (Fig. 5B–C), indicating denser cortical connections for rotated stimuli than non-rotated ones. In all sub-stages, CHANNEL showed significant main effects (all, p<0.001) and its interactions with GROUP were significant as well (all, p<0.014), indicating different patterns of 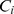 distribution in two groups. Control subjects showed larger 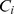 in frontal area (F3, F4, and Fz), while stroke patients showed larger 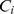 in left frontal area (Fp1 and F3) and right parietal area (P8, P4 and CP6) in all sub-stages (Fig. 5). Stroke patients had larger clustering coefficient than control subjects in contralesional (right) occipital (O2) and parietal (P8 and CP6) area in Beginning and Middle sub-stage (Fig. 5A–B). In End sub-stage, only contralesional parietal area (P8) showed significantly larger clustering coefficient in stroke patients than control subjects (Fig. 5C).

**Table 5:** ANOVA analysis of nodal clustering coefficient (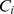).

	Beginning(0–300 ms)	Middle(300–800 ms)	End(800–1200 ms)
**GROUP**	F(1,20) = 0.938, p = 0.344	F(1,20) = 1.422, p = 0.247	F(1,20) = 0.001, p = 0.976
**HAND**	F(1,20) = 2.222, p = 0.152	F(1,20) = 3.688, p = 0.069	F(1,20) = 0.630, p = 0.437
**ANGLE**	F(5,100) = 14.523, p<0.001**	F(5,100) = 13.098, p<0.001**	F(5,100) = 9.474, p<0.001**
**CHANNEL**	F(27,540) = 3.042, p<0.001**	F(27,540) = 4.533, p<0.001**	F(27,540) = 15.088, p<0.001**
**GROUP × HAND**	F(1,20) = 0.864, p = 0.364	F(1,20) = 1.650, p = 0.214	F(1,20) = 1.146, p = 0.297
**GROUP × ANGLE**	F(5,100) = 1.885, p = 0.104	F(5,100) = 2.013, p = 0.083	F(5,100) = 1.021, p = 0.357
**GROUP × CHANNEL**	F(27,540) = 1.859, p = 0.006*	F(27,540) = 1.905, p = 0.004*	F(27,540) = 1.724, p = 0.014*

Significance was indicated by *(p<0.05) and **(p<0.001).

#### Nodal betweenness

**Figure 6:**
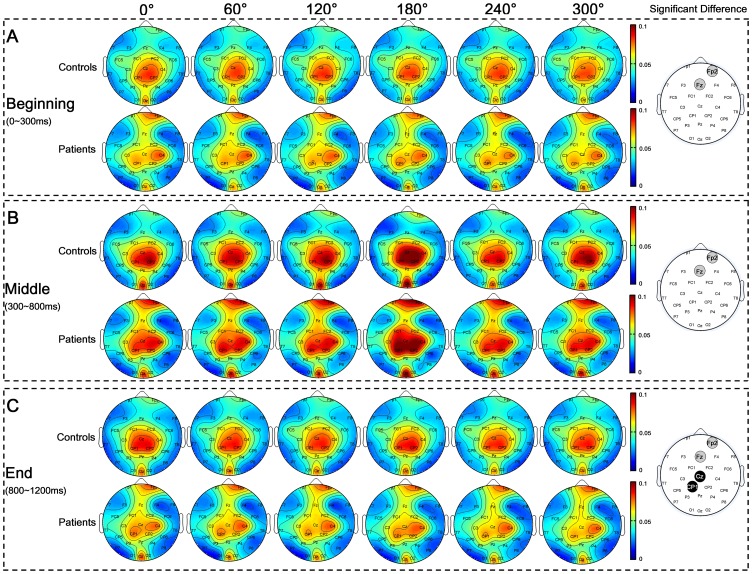
Nodal betweenness in three sub-stages. Nodal betweenness of two groups with respect to angle in Beginning (**A**), Middle (**B**), and End sub-stages (**C**) were illustrated respectively. Gray wafers indicated the channels where stroke patients have larger betweenness than control subjects, while black wafers indicated the channels where patients have lower betweenness than control subjects by t-test (p<0.05). However, the t-test p-values of these channels are larger than the significance threshold estimated by FDR (q<0.05) for multiple comparisons correction.

ANOVA results of nodal betweenness (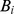) were shown in Table 6. In Beginning and End sub-stages, GROUP showed significant main effect that stroke patients had smaller betweenness than control subjects (F(1,20) = 6.776, p = 0.017; F(1,20) = 7.863, p = 0.011 respectively). However, in Middle sub-stage, no significant GROUP effect was observed (F(1,20) = 1.238, p = 0.279). Main effect of HAND was observed in all sub-stages (all, p<0.001) which was due to the fact that right hand stimulus had significantly larger betweenness than that for left hand stimulus. ANGLE also showed significant main effect (all, p<0.001), since nodal betweenness had larger value at 180° than other angles, particularly in Middle sub-stage. Main effects of CHANNEL (all, p<0.001) and its interactions with GROUP were also significant (all, p<0.027) in all sub-stages, which indicated different betweenness distribution patterns in two groups (Fig. 6). Control subjects showed larger betweenness in central area (Cz, CP1 and CP2) and occipital area (Oz) while stroke patients showed larger betweenness in right prefrontal area (Fp2), central area (Cz,C4,CP1 and CP2), and occipital (Oz) area. In all sub-stages, stroke patients had significantly larger betweenness in contralesional (right) prefrontal (Fp2) and frontal (Fz) area than control subjects (Fig. 6A–B). In addition, in response cognitive process, patients also showed significantly smaller betweenness in ipsilesional (left) central area (CP1 and Cz) than control subjects (Fig. 6*C*).

**Table 6:** ANOVA analysis of nodal betweenness (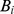).

	Beginning(0–300 ms)	Middle(300–800 ms)	End(800–1200 ms)
**GROUP**	F(1,20) = 6.776, p = 0.017*	F(1,20) = 1.238, p = 0.279	F(1,20) = 7.863, p = 0.011*
**HAND**	F(1,20) = 29.375, p<0.001**	F(1,20) = 53.140, p<0.001**	F(1,20) = 40.680, p<0.001**
**ANGLE**	F(5,100) = 61.661, p<0.001**	F(5,100) = 65.074, p<0.001**	F(5,100) = 62.855, p<0.001**
**CHANNEL**	F(27,540) = 2.522, p<0.001**	F(27,540) = 2.496, p<0.001**	F(27,540) = 3.186, p<0.001**
**GROUP × HAND**	F(1,20) = 1.811, p = 0.054	F(1,20) = 1.916, p = 0.182	F(1,20) = 1.725, p = 0.204
**GROUP × ANGLE**	F(5,100) = 1.769, p = 0.126	F(5,100) = 1.715, p = 0.138	F(5,100) = 1.198, p = 0.145
**GROUP × CHANNEL**	F(27,540) = 1.596, p = 0.030*	F(27,540) = 1.642, p = 0.023*	F(27,540) = 1.604, p = 0.029*

Significance was indicated by *(p<0.05) and **(p<0.001).

In short, stroke patients had larger nodal clustering coefficient and betweenness in contralesional occipitoparietal and frontal areas respectively in all sub-stages. In addition, lower betweenness in ipsilesonal central area in stroke patients was observed when they made response.

## Discussion

In this study, the alterations of functional brain network after stroke were examined from both global and nodal perspectives in three cognitive sub-stages during MRT. We found that: (i) Neural synchrony was impaired in stroke patients in mental rotation sub-stage. (ii) Functional brain networks of stroke patients demonstrated smaller global clustering coefficient and longer characteristic path length compared with control subjects in mental rotation sub-stage. (iii) Stroke patients had larger nodal clustering coefficient and betweenness in contralesional parietal and frontal area respectively in all sub-stages. In addition, lower betweenness of stroke patients in ipsilesional central area was observed in response sub-stage. In summary, network of stroke patients showed the reduction of neural synchrony and the alterations in both global and nodal properties of the cortical connectivity. The influence of stroke on functional network of motor imagery cognition would be discussed from these three perspectives.

### Reduction of Neural Synchrony

The larger angle that the visual stimulus was rotated, the larger PSI was found across the cortex, indicating higher synchronization level for more difficult task, i.e., “angle effect” in mental rotation sub-stage. Neural synchrony reduced significantly in stroke patients when they mentally rotated the hand pictures. Previous studies on different lesion locations also showed significant synchronization reduction in stroke patients, and strongly suggested that functional outcome after stroke could be predicated by how brain areas were coupled [30]–[31]. Patients showed smaller PSI when they mentally rotated the affected hand (right) picture than the unaffected hand, which might be due to the fact that the right hand is dominant for patients (presumably handness) and the impairment is more prominent than non-dominant left hand. In addition, during response sub-stage, patients showed larger neural synchrony for affected hand than unaffected hand, which might be related to greater recruitment of cortical areas [32]. These results could also imply a compensatory effect after stroke when they output actual movement.

### Alterations of Global Properties

Previous studies showed that the functional network after stroke had lower network efficiency or local connectedness during both resting-state and movement execution [9], [33]. Fallani et al. had investigated functional connectivity of stroke patients during preparation and execution in a finger tapping task. They found that the stroke patients’ capability to integrate information between distant brain regions significantly reduced and the number of disconnected nodes increased which were related with smaller clustering coefficient [9]. In this study, compared with healthy control subjects, the functional network of stroke patients showed lower local connective density (smaller clustering coefficient) and global network efficiency (longer characteristic path length) during MRT, especially when the visual stimulus was mentally rotated. All these results indicated that the topology of functional networks of both actual and imagery movements after stroke were significantly impaired and shifted from optimal small-world organization towards a random mode. The similarity of the alterations between imagery and actual movements might provide the neural network substrates for clinical stroke rehabilitation based on motor imagery training.

### Alterations of Nodal Properties

Nodal clustering coefficient represented the density that a node’s neighbors were connected, representing the functional segregation property of network. In this study, neuroplastic reorganization in contralesional hemisphere of stroke patients, i.e., compensatory effect of contralesional occipitoparietal cortex on the functional segregation, was observed. In a fMRI study of chronic stroke patients, Schaechter *et al.* found both skilled and unskilled movement elicited more activations in patients than control subjects in the contralesional primary sensorimotor cortex, ventral premotor cortex, supplementary motor area and occipitoparietal cortex, which implied a similar neuroplastic reorganization in contralesional hemisphere [34]. Gerloff *et al.* also observed increase of contralesional activity, from both focal activation and connectivity perspectives by EEG, and they considered the increase of contralesional activities might facilitate the control of recovered motor function at a higher-order processing level [35]. In this study, for motor imagery task, contralesional occipitoparietal cortex showed larger clustering coefficient after stroke, which might also be attributed to the neuroplastic reorganization to facilitate the MRT. All these results showed contralesional occipitoparietal compensatory effect on local activation and connectivity in both actual movement and motor imagery, which also provided the cognitive substrates for stroke rehabilitation based on motor imagery training.

Nodal betweenness reflected the importance and centrality of a node in the information transfer over whole network. Larger betweenness meant more shortest paths passed through the corresponding node and greater centrality in global information transfer over the whole network [8]. In this study, stroke patients showed larger node centrality in contralesional prefrontal and frontal area in Beginning and Middle sub-stages. Previous study also found a prefrontal compensatory reorganization in unaffected hemisphere in stroke patients [36]–[37]. In this study, in the first two sub-stages of MRT, stroke patients had to recruit contralesional prefrontal brain area with larger centrality to encode hand pictures and mentally rotate them. In response cognitive process, smaller ipsilesional central-parietal node centrality indicated the impaired information transfer in left hemispheric movement-related areas. Using diffusion MRI tractography, Crofts *et al*. also investigated the “communicability” of stroke patients, which indicated the ability of information transfer across network. They found reduced ipsilesional communicability in regions surrounding the lesions [38]. Therefore, the impaired node centrality in ipsilesional hemisphere after stroke also should be considered, from the perspective of the efficiency in neural network information transfer, in designing rehabilitation strategy based on motor imagery.

### Study Limitation

In this study, only 28-channel EEG data from eleven patients were used to investigate stroke lesion impact on functional network during MRT. Besides, no neuropsychological test was administered to investigate putative impairments in body representation. Although all patients were selected with stroke in left hemisphere, the lesion locations were not at exactly the same location, otherwise, the number of the subjects would be too few to have statistical analysis. To understand the role of a specific region of brain on the cortical functional network for motor imagery cognitive process, a larger sample size and more stringent restrictions of lesion locations should be considered, which would be our next step study.

## Conclusion

This study provided the details of functional brain network alterations after ischemic stroke in each cognitive sub-stage during motor imagery. In summary, neural synchrony during motor imagery task was impaired after stroke. Stroke patients reflected a lower capability to integrate the communication between widely separated brain regions and lower tendency to have dense local connectedness in brain networks. Stroke patients also showed neuroplastic reorganization in contralesional hemisphere.
